# Supplementary material for: Dynamic transcriptomic profiles of zebrafish gills in response to zinc supplementation
Source: BMC Genomics. 2010 Oct 11;11:553. doi: 10.1186/1471-2164-11-553 (PMC3091702; doi:10.1186/1471-2164-11-553)
Supplement: Additional file 2 — Interactive Direct Interaction Network representing the molecular interactions between zinc, copper, iron, calcium and proteins encoded by transcripts changed by zinc supplementation. Mini web-site containing index.html and hyperlinked pages in subdirectory describing a Direct Interaction Network automatically generated based on curated interactions contained within the proprietary PathwayArchitect database. Ovals represent proteins and the circles symbolize metal ions. Objects are coloured by their abundance in zebrafish at the time-point they were significantly different from the control is a scale from -4 fold (dark green) to +4 fold (dark red). Where significant differences were found at more than one time-point, the colour overlay shows expression at the first instance. Dark blue squares denote 'binding', and light blue squares 'expression'; green squares stand for 'regulation', green diamonds for 'metabolism', and green circles for 'promoter binding'. Arrow heads indicate directionality of the interaction where annotated. All nodes and edges can be further interrogated by selecting the relative area of the image. [file 1471-2164-11-553-S2.zip › PathwayArchitect Zn xs DIN/607474.html]

# REGULATION:

|  |  |
| --- | --- |
| Type | REGULATION |
| Effect | Negative |


---

|  |  |
| --- | --- |
| Score | 0 |


---

|  |  |
| --- | --- |
| Reference Count | 4 |


---

|  |  |
| --- | --- |
| Mechanism | Unknown |


---

|  |  |
| --- | --- |
| Reference:0 || Sentence | "The iron chelators desferrioxamine (DFO), 1,2-dimethyl(L1)-, 1-ethyl-2-methyl(L1NEt)- and 1-propyl-2-methyl(L1NPr)-3-hydroxypyrid-4-ones inhibited rat aortic prostacyclin (PGI2) synthesis in vitro (rank order of potency: DFO greater than L1 greater than L1NEt greater than L1NPr) when stimulated with adrenaline, arachidonate and the Ca2+ ionophore A23187." |
| PMID | 3140797 |
| Year | 1988 |
| Species | Rat |
| Journal | Biochem J |
| RefScore | 0 |
| Source | PArchNLP |
  |
|  |  |


---

|  |  |
| --- | --- |
 Reference:1 || Sentence | "An iron complex concentration-dependent inhibition of the CaATPase activity, and its consequent Ca2+-extrusion impairment appear as the likely cause of calcium overload." |
| PMID | 9852309 |
| Year | 1998 |
| Journal | Int J Mol Med |
| RefScore | 1 |
| Source | PArchNLP |
  ||  |  |


---

|  |  |
| --- | --- |
 Reference:2 || Sentence | "Ca2 + reperfusion injury induced by Ca2 + depletion or H2O2 exposure was blocked by the iron chelator 1, 10-phenanthroline, the NF-kappaB inhibitor pyrrolidinedithiocarbamate and the calcineurin inhibitor FK506." |
| PMID | 10594646 |
| Year | 1999 |
| Species | Rat |
| Journal | Eur J Neurosci |
| RefScore | 1 |
| Source | PArchNLP |
  ||  |  |


---

|  |  |
| --- | --- |
 Reference:3 || Sentence | "Cell death is now mainly mediated by iron: the iron chelators and hydroxyl radical scavengers prevented cell injury, the increase in cytosolic Ca2+ was significantly less pronounced, and BAPTA/AM did not exert any protection under these conditions." |
| PMID | 9741585 |
| Year | 1998 |
| Species | Mouse |
| Journal | Free Radic Biol Med |
| RefScore | 1 |
| Source | PArchNLP |
  |


---

|  |  |
| --- | --- |
